# Inhibition of the IgE-Mediated Activation of RBL-2H3 Cells by TIPP, a Novel Thymic Immunosuppressive Pentapeptide

**DOI:** 10.3390/ijms16012252

**Published:** 2015-01-20

**Authors:** Qianqian Lian, Yanna Cheng, Chuanqing Zhong, Fengshan Wang

**Affiliations:** 1 Key Laboratory of Chemical Biology of Natural Products (Ministry of Education), Institute of Biochemical and Biotechnological Drugs, School of Pharmaceutical Sciences, Shandong University, Jinan 250012, China; E-Mails: xhrg_1042@126.com (Q.L.); zhongchq@163.com (C.Z.); 2 Department of Pharmacology, School of Pharmaceutical Sciences, Shandong University, Jinan 250012, China; E-Mail: chengyn@sdu.edu.cn; 3 National Glycoengineering Research Center, Shandong University, Jinan 250012, China

**Keywords:** allergy, IgE, RBL-2H3 cell, MAP kinases, NF-κB, TIPP

## Abstract

TIPP is a novel thymic immunosuppressive pentapeptide originally obtained from calf thymic immunosuppressive extract. The present study aimed to investigate the inhibitory activity of TIPP on IgE-mediated activation of RBL-2H3 cells. Release of β-hexosaminidase and histamine, intracellular calcium, membrane ruffling, mRNA levels of cytokines, cyclooxygenase-2 (COX-2) expression, and activation of mitogen-activated protein kinases (MAP kinases) and NF-κB were determined by colorimetric assay, fluorescence spectrophotometer, confocal fluorescence microscope, quantification PCR, and Western blot, respectively. The results showed that TIPP significantly inhibited the degranulation in IgE-antigen complex-stimulated RBL-2H3 cells without cytotoxicity. TIPP significantly suppressed the increase of intracellular calcium and the rearrangement of F-actin, attenuated the transcription of pro-inflammatory cytokines (IL-3, -4, -6, -13, TNF-α, and monocyte chemotactic protein-1 (MCP-1)), and decreased the expression of COX-2. Western blot analysis showed that TIPP had an inhibitory activity on the phosphorylation of extracellular signal-regulated protein kinase 1/2 (ERK1/2) and ERK kinase 1/2 (MEK1/2), and inhibited the activation of NF-κB. The data suggested that TIPP effectively suppressed IgE-mediated activation of RBL-2H3 cells via blocking MEK/ERK and NF-κB signaling pathways.

## 1. Introduction

As a major part of the immune system, mast cells play an important role in innate and adaptive immunity, especially in allergic and inflammatory responses [1,2]. Mast cells express a high-affinity IgE receptor, FcεRI, on membranes, and the binding of IgE-antigen complexes to FcεRI triggers complex biological reactions. Degranulation is preceded by increased Ca^2+^ influx, with the release of inflammatory mediators contained in the cytoplasmic granules including histamine, cytokines, leukotrienes, and many proteases [1]. In addition, mast cell activation results in cytoskeletal reorganization and a series of intracellular signaling molecular activations [3,4]. Newly generated inflammatory mediators, such as cytokines and chemokines, are produced during the activation as well. The releasing mediators, including preformed and newly generated ones, have profound effects. For example, histamine is a hallmark of allergic responses and induces vascular permeability, while the secreted cytokines and chemokines play a pivotal role in initiating and maintaining inflammatory responses in allergic reactions [2].

**Figure 1:**
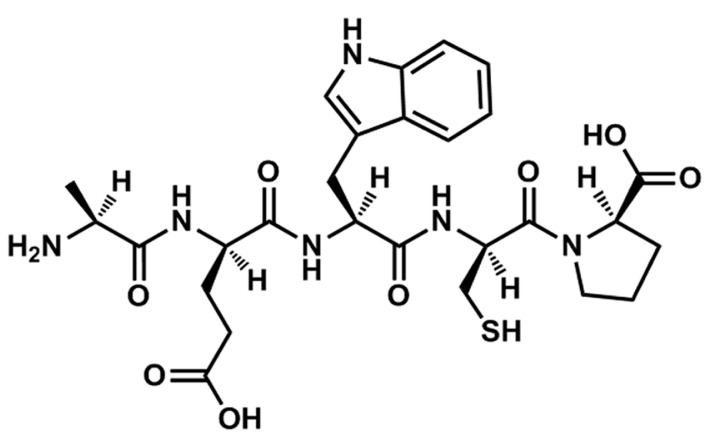
Chemical structure of TIPP.

TIPP is a novel thymic immunosuppressive pentapeptide originally obtained from calf thymic immunosuppressive extract (TISE). It was reported that there were non-cytotoxic-specific inhibitors (termed as “thymic chalones” or “lymphocyte chalones”) in animal thymic extracts [5,6,7]. Experimental results showed that these crude extracts had negative regulating functions in the DNA synthesis and proliferation of lymphocytes [8,9]. Some experiments had been done to explain the composition and the chemical nature of thymic chalones. Houck* et al.* presented evidence that thymic chalone was thermolabile and trypsin-labile, and had a mass of 30–50 kD [5,8]. Kiger* et al.* reported that the active molecule seemed to be a heat-resistant basic peptide, probably bound to a ribonucleotide moiety [7]. Allen* et al.* reported that the activity moiety of chalone was identified biologically and chemically as spermine, and spermine complex was formed in thymic extracts with an unidentified tissue-specific material acting as a carrier for spermine [6]. Maschler and Maurer isolated a fraction of chalones, with a molecular weight below 1400, and found that the fraction could inhibit the growth of lymphocyte colonies, an activity unlikely to result from spermine [10]. Patt* et al.* isolated a fraction that appeared to be a polypeptide that had an estimated molecular weight of 500–600 and was heat and pH stable [11]. There were also many other studies focused on the immune inhibitory factors, but no component with identified structure was reported. Our lab began to study TISE in the 1980s and developed a new method for TISE preparation. Our prior research has shown that TISE inhibited the immune and allergic responses effectively both* in vitro* and* in vivo* [12,13,14]. After further isolation and purification, a novel pentapeptide with the sequence of Ala-Glu-Trp-Cys-Pro (TIPP, Figure 1) was obtained from TISE originating from calf thymus. In view of the apparent effects of TISE against allergic responses, we speculated that TIPP may have similar activity. Thus, we focused on investigating the anti-allergic activity and mechanism of TIPP with rat basophilic leukemia cells (RBL-2H3)* in vitro* in this study.

## 2. Results

### 2.1. Cytotoxicity of Thymic Immunosuppressive Pentapeptide (TIPP) on RBL-2H3 Cells

**Figure 2:**
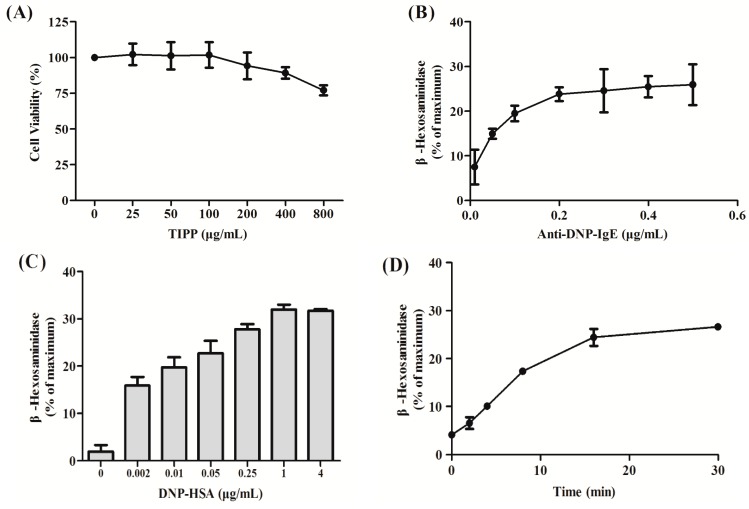

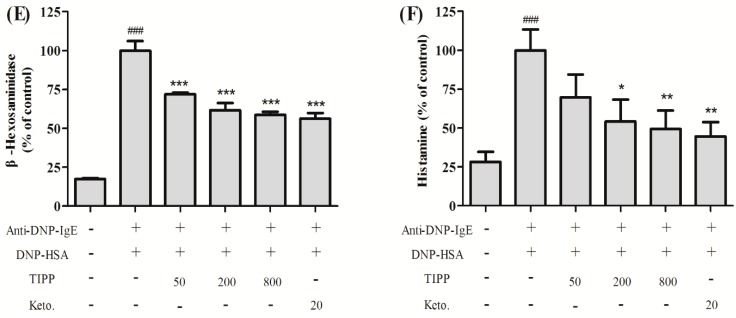
Effect of thymic immunosuppressive pentapeptide (TIPP) on IgE-mediated degranulation in RBL-2H3 cells. (**A**) Cytotoxicity of TIPP. Results are expressed as mean ± SD (*n =* 5); (**B**–**D**) represent the effects of anti-DNP-IgE (monoclonal anti-dinitrophenyl antibody produced in mouse, IgE isotype) concentration, dinitrophenyl-human serum albumin (DNP-HAS) concentration, and stimulated time on IgE-mediated degranulation in RBL-2H3 cells. The amount of β-hexosaminidase in culture supernatant was determined as a biomarker of degranulation. Supernatant samples treated with 0.1% Triton X-100 (*v*/*v*) were used as a maximum of degranulation. Results are expressed as mean ± SEM (*n =* 3); (**E**,**F**) represent the effects of TIPP on β-hexosaminidase and histamine release under optimum conditions. Supernatant samples stimulated with IgE-antigen complex and not treated with TIPP were used as a control of 100%. Results are expressed as mean ± SEM (*n =* 3 for β-hexosaminidase determination and *n =* 6 for histamine determination). Compared to normal, ^###^* p* < 0.001; compared to control (sensitized with anti-DNP-IgE and stimulated with DNP-HSA), * p < 0.05, ** p < 0.01, and *** p < 0.001. Keto.: ketotifen.

In order to evaluate the cytotoxicity of TIPP on RBL-2H3 cells, the cells were treated with different concentrations of TIPP for 24 h and the MTT (3-(4,5-dimethylthiazol-2-yl)-2,5-diphenyl-tetrazolium bromide) method was used for the cytotoxicity assay. As shown in Figure 2A, TIPP had no significant cytotoxicity on RBL-2H3 cells.

### 2.2. Optimum Conditions for IgE-Mediated Degranulation in RBL-2H3 Cells

As shown in Figure 2B–D, IgE-mediated degranulation in RBL-2H3 cells was a dose- and time-dependent process. Optimum conditions for IgE-mediated degranulation in RBL-2H3 cells were sensitizing with 0.2 μg/mL anti-DNP-IgE (monoclonal anti-dinitrophenyl antibody produced in mouse, IgE isotype) and stimulating with 1 μg/mL DNP-HSA (dinitrophenyl-human serum albumin) for 15 min.

### 2.3. Effect of TIPP on the Release of β-Hexosaminidase and Histamine in IgE-Antigen Complex-Stimulated RBL-2H3 Cells

The inhibitory effect of TIPP on IgE-mediated degranulation in RBL-2H3 cells was measured with β-hexosaminidase and histamine secretions as degranulation biomarkers (Figure 2E,F). The releases of β-hexosaminidase and histamine in IgE-antigen complex-stimulated RBL-2H3 cells were significantly increased compared with normal cells. Pre-treatment with 20 μg/mL of ketotifen (Keto., an anti-allergic agent) significantly suppressed the degranulation in IgE-antigen complex-stimulated RBL-2H3 cells. In addition, TIPP treatment reduced the degranulation in RBL-2H3 cells stimulated with IgE-antigen complex in a dose-dependent manner.

### 2.4. Effect of TIPP on the Intracellular Calcium Level

**Figure 3:**
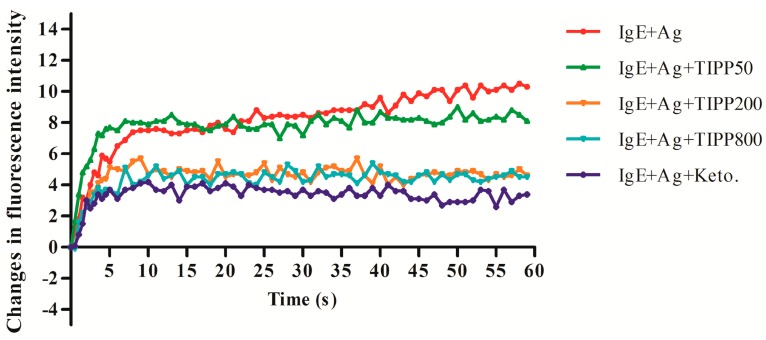
Effect of TIPP on intracellular calcium level. This result is representative of three independent experiments with similar results.

The inhibitory effect of TIPP on the intracellular calcium level of activated RBL-2H3 cells was detected with Fluo 3-AM ((4-(6-acetoxymethoxy-2,7-dichloro-3-oxo-9-xanthenyl)-4'-methyl-2,2'(ethylenedioxy)dianiline-*N*,*N*,*N*',*N*'-tetraacetic acid tetrakis(acetoxymethyl) ester)), a calcium-specific fluorescent probe. As shown in Figure 3, antigen stimulation on sensitized RBL-2H3 cells caused an increase in the fluorescence intensity changes, which indicated that the intracellular calcium level was increased by antigen stimulation. TIPP (200 and 800 μg/mL) and ketotifen pretreatments could suppress the intracellular calcium level changes caused by antigen as the results showed decreased changes in fluorescence intensity in treated groups.

### 2.5. Effects of TIPP on the mRNA Levels of Pro-Inflammatory Cytokines

**Figure 4:**
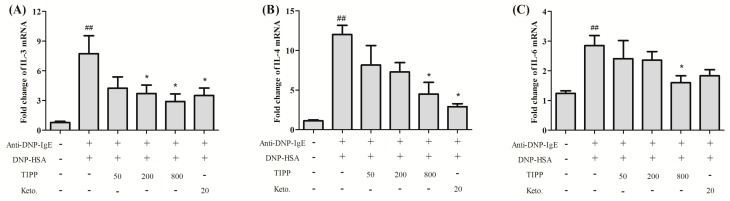

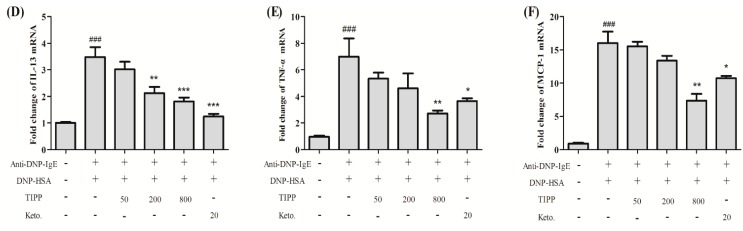
Effects of TIPP on the mRNA levels of (**A**) IL-3; (**B**) IL-4; (**C**) IL-6; (**D**) IL-13; (**E**) TNF-α; and (**F**) monocyte chemotactic protein-1 (MCP-1) in IgE-antigen complex-stimulated RBL-2H3 cells. mRNA level of β-actin was used as an internal control. Results are expressed as mean ± SEM (*n =* 4). Compared with normal group, ^##^ *p* < 0.01, ^###^ *p* < 0.001; compared with control group (sensitized with anti-DNP-IgE and stimulated with DNP-HSA), * *p* < 0.05, ** *p* < 0.01, and *** *p* < 0.001.

The inhibitory effects of TIPP on pro-inflammatory cytokine production were assessed by fluorescent quantitative PCR. Activation of RBL-2H3 cells by an IgE-antigen complex improved the mRNA levels of interleukins (IL-3, -4, -6, and -13), monocyte chemotactic protein-1 (MCP-1), and TNF-α; and pre-treatment with TIPP before stimulation decreased their levels in activated RBL-2H3 cells (Figure 4).

### 2.6. The Inhibitory Effect of TIPP on Membrane Ruffling

**Figure 5:**
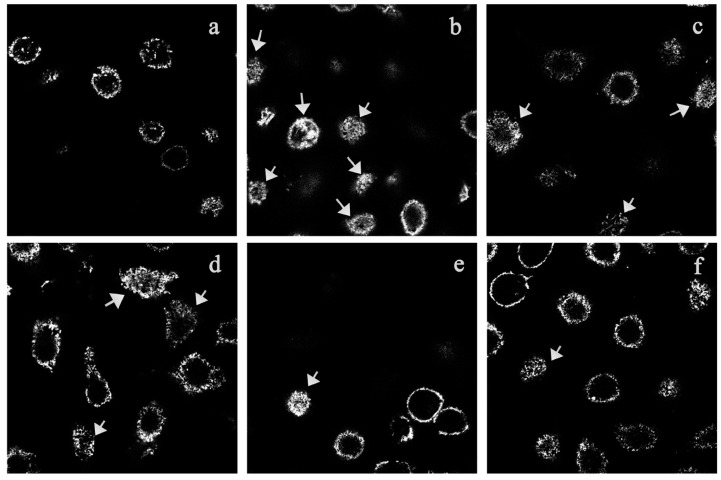
Confocal fluorescence microscope observation of F-actin (labeled with rhodamin-phalloidin) in RBL-2H3 cells. The arrow represents membrane ruffling caused by F-actin rearrangement. (**a**) IgE-sensitized RBL-2H3 cells stimulated with PBS; (**b**) IgE-sensitized RBL-2H3 cells stimulated with DNP-HSA for 30 min; (**c**–**f**), IgE-sensitized RBL-2H3 cells, pretreated with 50, 200, and 800 μg/mL of TIPP or 20 μg/mL of ketotifen, then stimulated with DNP-HSA for 30 min. Magnification: 63×.

As shown in Figure 5, F-actin is neatly arranged around the periphery of the normal RBL-2H3 cells (Figure 5a). Stimulation with DNP-HSA antigen caused a remarkable rearrangement of F-actin, which resulted in a membrane ruffling (Figure 5b). Membrane ruffling was decreased apparently and F-actin tended to be neatly arranged when RBL-2H3 cells were pre-treated with 800 μg/mL of TIPP or 20 μg/mL of ketotifen (Figure 5e,f).

### 2.7. The Interaction of TIPP and RBL-2H3 Cells

**Figure 6:**
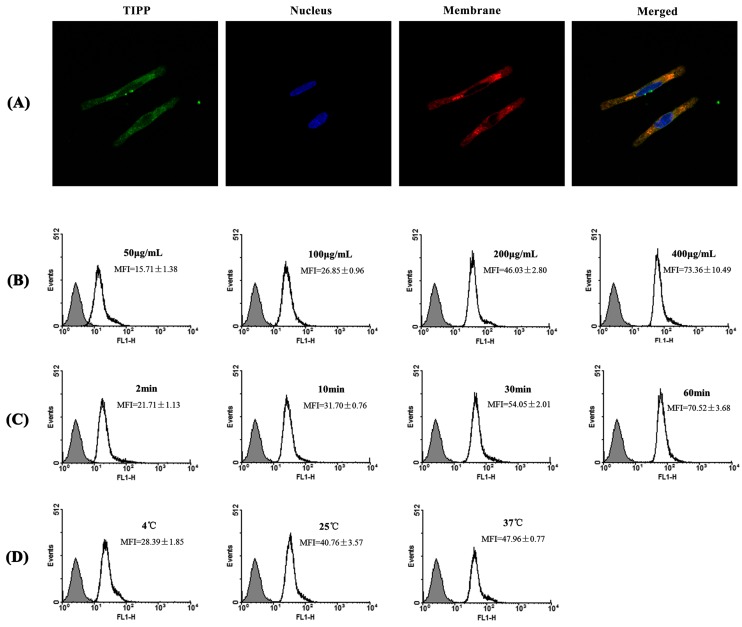
(**A**) Confocal microscopic observation of the binding of TIPP with RBL-2H3 cells. Pictures represent location of TIPP, nucleus, membrane and merged, respectively. Magnification: 63×; (**B**–**D**) represent the concentration-, time- and temperature-dependent characterization of the TIPP binding.

The mode of TIPP binding with RBL-2H3 cells was tested. The confocal fluorescence microscope results showed that TIPP bound with the cell membrane and could get into cells (Figure 6A). The binding kinetics was determined by flow cytometry analysis. A higher mean fluorescent intensity was obtained with increases in TIPP concentration, time, and temperature, which indicated the effect of concentration-, time- and temperature-dependence in the binding between TIPP and RBL-2H3 cells (Figure 6B–D).

### 2.8. The Inhibitory Effect of TIPP on Cyclooxygenase-2 (COX-2) Expression

**Figure 7:**
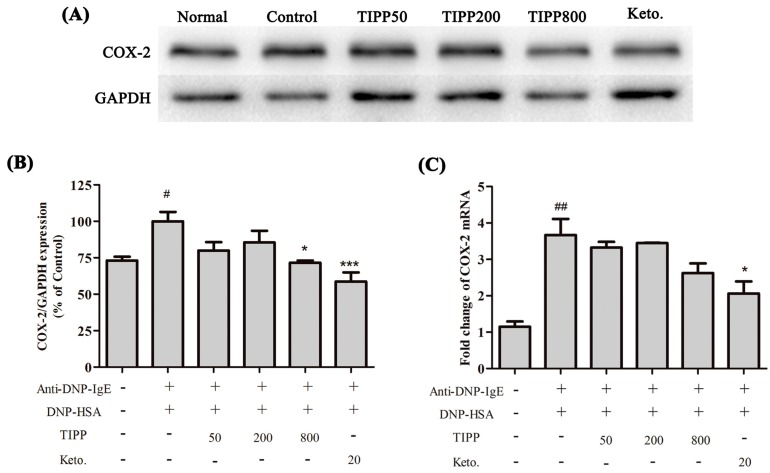
Western blot diagram (**A**) and statistical analysis (**B**) of COX-2 (cyclooxygenase-2) expression in IgE-antigen complex stimulated RBL-2H3 cells; (**C**) represents the statistical analysis of COX-2 mRNA levels in IgE-antigen complex stimulated RBL-2H3 cells. The Western blot diagram is a representative of three independent experiment diagrams with similar results. Each lane was loaded with 20 μg of total protein. Results are expressed as mean ± SEM (*n* = 3). Compared with normal group, ^#^ *p* < 0.05, ^##^ *p* < 0.01; compared with control group, * *p* < 0.05, *** *p* < 0.001.

Sensitized RBL-2H3 cells were stimulated for 1 h to detect cyclooxygenase-2 (COX-2) expression. The statistic result showed that COX-2 expression was enhanced in RBL-2H3 cells activated by antigen-IgE complex, and TIPP (800 μg/mL) or ketotifen pre-treatment significantly inhibited the protein expression in activated RBL-2H3 cells (Figure 7).

### 2.9. Effects of TIPP on Mitogen-Activated Protein (MAP) Kinase Activation

**Figure 8:**
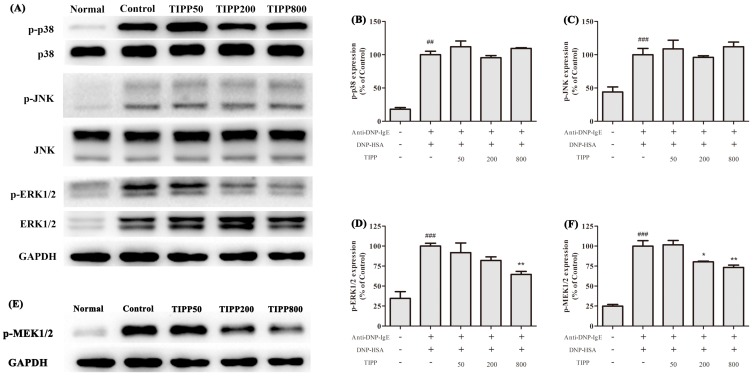
Effects of TIPP on the activation of mitogen-activated protein (MAP) kinases (**A**–**D**) and extracellular signal-regulated protein kinase (ERK) kinase 1/2 (MEK1/2) (**E**,**F**) in IgE-antigen complex stimulated RBL-2H3 cells. The Western blot diagram is a representative of three independent experiment diagrams with similar results. Each lane was loaded with 20 μg of total protein. Results are expressed as mean ± SEM (*n =* 3). Compared with Normal group, ^##^ *p* < 0.01, ^###^ *p* < 0.001; compared with Control group, * *p* < 0.05, ** *p* < 0.01.

Mitogen-activated protein (MAP) kinase (extracellular signal-regulated protein kinase (ERK), *c*-Jun *N*-terminal kinase (JNK), and p38 mitogen-activated protein kinase (p38)) pathways have been identified to reflect RBL-2H3 activation. Thus, the levels of phosphorylation of three MAP kinases were examined. The results showed that the antigen stimulation caused apparent increases in the phosphorylation of the three MAP kinases compared with normal cells. TIPP treatment significantly reduced the upregulation of phosphorylation of ERK in activated RBL-2H3 cells but showed no effect on the levels of phosphorylation of p38 and JNK (Figure 8A–D).

### 2.10. Effect of TIPP on Extracellular Signal-Regulated Protein Kinase (ERK)/ERK Kinase (MEK) Signaling Pathway

As MEK1/2 are specific kinases that mediate the activation of extracellular signal-regulated protein kinase 1/2 (ERK1/2), we detected the effect of TIPP on the level of phosphorylation of MEK1/2. As illustrated in Figure 8E,F, TIPP treatment (200 and 800 μg/mL) significantly inhibited the phosphorylation of MEK1/2 in the IgE-antigen activated RBL-2H3 cells.

### 2.11. Effect of TIPP on the Nuclear Translocation of NF-κB

**Figure 9:**
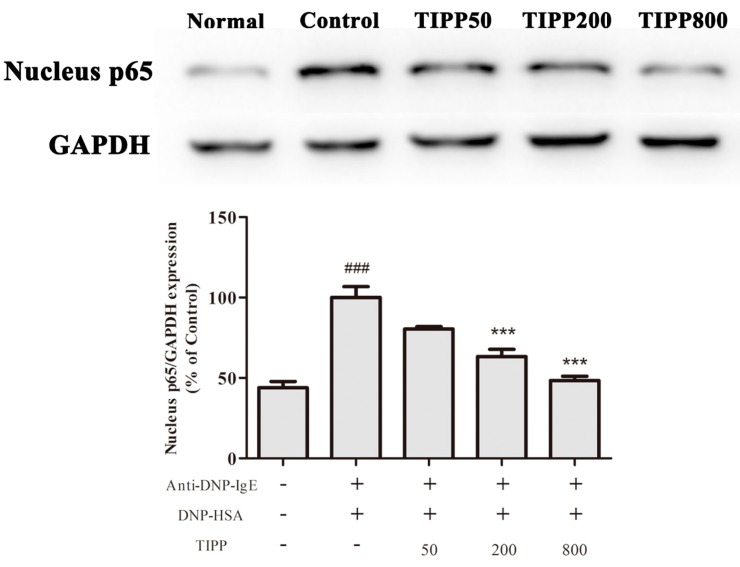
Effect of TIPP on the nuclear translocation of NF-κB. The Western blot diagram is a representative of three independent experiment diagrams with similar results. Each lane was loaded with 20 μg of total protein. Results are expressed as mean ± SEM (*n =* 3). Compared with Normal group, ^###^ *p* < 0.001; compared with Control group, *** *p* < 0.001.

To determine whether TIPP influenced NF-κB activation, the level of nuclear NF-κB in RBL-2H3 cells was analyzed by Western blot. As illustrated in Figure 9, the level of p65, a subunit of NF-κB, was increased in the nucleus of IgE-antigen complex-stimulated RBL-2H3 cells, and TIPP pre-treatment showed an inhibitory activity on the nuclear translocation of NF-κB. The results indicated that TIPP inhibited the activation of NF-κB caused by IgE-antigen complex stimulation in RBL-2H3 cells.

## 3. Discussion

Mast cells are multifunctional cells and have important impacts on the initiation and development of an allergic response. They can be activated by a series of stimuli, through IgE-dependent or independent mechanisms, releasing and producing inflammatory mediators [1]. The rat basophilic leukemia cells, RBL-2H3, are a tumor analogue of mast cells with a high expression of FcεRI on the cell surface, and can be activated by the IgE-antigen complex. In this study, we used RBL-2H3 cells to investigate the anti-allergic activity of TIPP* in vitro*. RBL-2H3 cells can release histamine and various inflammatory cytokines in the process of degranulation when activated via an IgE-antigen complex bound to FcεRI. In our research, we found that TIPP inhibited the secretion of β-hexosaminidase and histamine in IgE-antigen complex-stimulated RBL-2H3 cells, which indicated the inhibitory activity of TIPP to mast cell degranulation.

Calcium movement is closely related to the mechanism of mast cell degranulation [15]. An increase in the level of intercellular calcium [Ca^2+^]_i_ occurred when a mast cell is activated. Inhibition on the changes of [Ca^2+^]_i_ level contributes to the attenuation of the degranulation. Our results showed an inhibitory effect of TIPP on [Ca^2+^]_i_ level in IgE-antigen complex-activated RBL-2H3 cells, which suggested the inhibitory effect of TIPP on degranulation.

Inflammatory cytokines mediate the pathological reaction in inflammatory diseases. Besides the secretion of stored cytokines during degranulation, the production of newly generated inflammatory cytokines (IL-4, IL-6, IL-13, TNF-α,* etc.*) is induced [1]. The inducible cyclooxygenase COX-2, a marker of inflammation, is induced to be expressed in the activated mast cells. We assessed the activity of TIPP on the mRNA levels of IL-3, IL-4, IL-6, IL-13, TNF-α, and MCP-1 and the protein expression of COX-2 in IgE-antigen complex-activated RBL-2H3 cells. Treatment with TIPP before stimulation suppressed the increases of the mRNA levels in IgE-activated cells, and decreased the expression of COX-2 as well. These results indicated the anti-allergic property of TIPP in allergic inflammation therapy.

A series of dynamic morphological changes occur when RBL-2H3 cells are stimulated by IgE-antigen complex. The membrane appears to be pleated and ruffled. As an important cytoskeleton protein, F-actin is closely related to membrane changes. It will be concentrated and rearranged in the cell surface when the cells are activated [16]. In our study, the results from fluorescence microscopy examination of the rhodamin-phalloidin-labeled cells showed TIPP treatment inhibited F-actin rearrangement in IgE-activated RBL-2H3 cells, and we believe that this inhibitory effect was a reflection of the anti-allergic activity of TIPP.

The activation of RBL-2H3 cells triggered by aggregation of FcεRI is regulated by complex series of intracellular signaling process [17,18]. MAPK cascade is one of the most important pathways involved in the immune response and regulates the expression of a series of mediators associated with inflammation [19]. Previous research has shown that three MAP kinases—ERK1/2, JNK1/2, and p38—are activated in the IgE-antigen complex-stimulated mast cells and are good targets for the pharmacological treatment of allergic inflammation [19,20,21]. In our study, TIPP had a negative effect on the transcription of inflammatory cytokines (IL-4, IL-6, IL-13, TNF-α,* etc.*) and also suppressed the expression of COX-2. Since MAP kinases play significant roles in the transcription and expression of these mediators, we assessed the effects of TIPP on the MAP kinase activation. It was shown that the antigen stimulation induced phosphorylation of ERK1/2, JNK1/2, and p38. Treatment with TIPP inhibited the phosphorylation of ERK1/2, but showed no effect on the activation of JNK1/2 and p38. Activation of ERK1/2 requires phosphorylation at Tyr204/187 and then Thr202/185, which is catalyzed by the dual specificity kinases MEK1/2 [22]. TIPP significantly inhibited the upregulation of phosphorylation of MEK1/2 in the antigen activated RBL-2H3 cells. These results indicated that TIPP showed an inhibitory effect on the IgE-mediated activation of RBL-2H3 cells via the MEK/ERK signaling pathway.

Nuclear factor-κB (NF-κB), a major transcription factor, regulates the transcription of multiple inflammatory and immune genes and plays a critical role in allergic and inflammatory diseases [23,24]. Evidence showed the activation of NF-κB in the activated mast cells and NF-κB participated in the mRNA transcription of IL-3, -4, -6, -13, TNF-α, MCP-1, and COX-2. To determine whether TIPP influenced NF-κB activation, we analyzed the NF-κB p65 protein level in nuclear extracts. The protein level of p65 in nuclear extracts was increased in the IgE-antigen complex-activated RBL-2H3 cells, and TIPP inhibited this upregulation in a dose-dependent manner. This result strongly suggested that TIPP reduced the inflammatory mediator production through inhibiting the activation of NF-κB.

## 4. Experimental Section

### 4.1. Reagents

TIPP (>95% purity) was synthesized by ChinaPeptides Co., Ltd. (Shanghai, China). Minimum Essential Medium (MEM) and fetal bovine serum (FBS) were purchased from Gibco (Paisley, UK). Anti-DNP-IgE (monoclonal anti-dinitrophenyl antibody produced in mouse, IgE isotype, clone SPE-7), DNP-HSA (dinitrophenyl-human serum albumin), 3-(4,5-dimethylthiazol-2-yl)-2,5-diphenyl-tetrazolium bromide (MTT), dimethyl sulfoxide (DMSO), *p*-nitrophenyl-*N*-acetyl-β-d-glucosaminide, *o*-phthalaldehyde, and Fluo 3-AM were purchased from Sigma-Aldrich (St. Louis, MO, USA). TRIzol reagent from Invitrogen (Carlsbad, CA, USA), FastQuant RT Kit from Tiangen Biotech Co., Ltd. (Beijing, China), and SYBR^®^ Green Real-time PCR Master Mix from Toyobo Co., Ltd. (Osaka, Japan) were used for quantification PCR analysis. Total protein extraction kit and nuclear protein extraction kit were purchased from BestBio (Shanghai, China). Anti-COX-2 and anti-phospho-ERK, anti-phospho-JNK, anti-phospho-p38, anti-NF-κB p65, anti-ERK, anti-JNK, anti-p38, anti-β-actin antibodies, and secondary antibodies (anti-rabbit or anti-mouse) were obtained from Cell Signaling Technology (Danvers, MA, USA). Immobilon™ Western chemiluminescent HRP substrate was produced by Millipore Corporation (Billerica, MA, USA).

### 4.2. Cell Culture

RBL-2H3 cells were obtained from the Cell Resources Center of the Shanghai Institutes for Biological Sciences, Chinese Academy of Sciences (Shanghai, China) and cultured in MEM with 15% heat-inactivated FBS at 37 °C in a humidified incubator (5% CO_2_, 95% air).

### 4.3. Cytotoxicity Assay of TIPP on RBL-2H3 Cells

RBL-2H3 cells were harvested and transferred into 96-well microplates (1 × 10^4^ cells/well) and cultured with TIPP of different concentrations (0–800 μg/mL) for 24 h at 37 °C in a humidified incubator (5% CO_2_, 95% air). Then, 20 μL of 5 mg/mL MTT was added and the cells were incubated for another 4 h at 37 °C. The precipitate was dissolved in DMSO and the absorbance was measured at 570 nm with a microplate reader (Bio-Rad 680, Hercules, CA, USA).

### 4.4. Cell Sensitization and Stimulation for Degranulation Assay

For the degranulation assay, cells were loaded onto 24-well microplates (2 × 10^5^ cells/0.4 mL/well), and incubated with 0.2 μg/mL of anti-DNP-IgE overnight for cell sensitization [21]. After washing with PBS three times, the cells were exposed to different concentrations of TIPP (0–800 μg/mL) or ketotifen (20 μg/mL) in PIPES (1,4-piperazinebis(ethanesulfonic acid)) buffer (119 mM NaCl, 5 mM KCl, 25 mM PIPES, 5.6 mM glucose, 1 mM CaCl_2_, 0.4 mM MgCl_2_, and 0.1% BSA, pH 7.2) for 30 min, and then stimulated with 1 μg/mL of DNP-HSA for 15 min. β-Hexosaminidase activity and histamine content in culture supernatants were measured as indicators of degranulation.

### 4.5. β-Hexosaminidase Activity and Histamine Content Determination

A colorimetric assay was used to determine the amount of β-hexosaminidase released from RBL-2H3 cells according to [25], with slight modification. Twenty-five microliters of cell culture supernatant were incubated with an equal volume of 5 mM substrate solution (5 mM *p*-nitrophenyl-*N*-acetyl-β-d-glucosaminide dissolved in 0.2 M sodium citrate buffer, pH 4.5) at 37 °C for 1.5 h. Then, the enzyme reaction was terminated by adding 200 μL of stop solution (0.1 M Na_2_CO_3_/NaHCO_3_, pH 10.0) and the absorbance was measured at 405 nm with a microplate reader.

In order to measure the amount of histamine released from RBL-2H3 cells, 20 μL of 1 M NaOH was added to 100 μL of cell culture supernatant, and then 25 μL of the reaction solution (1% (*w*/*v*) *o*-phthalaldehyde dissolved in methanol) was immediately added and mixed. After incubation at room temperature for 4 min (away from light), the reaction was terminated by the addition of 10 μL of 3 M HCl and the amount of histamine was determined using a fluorometric method [26]. The fluorescence intensity was measured at the excitation wavelength 355 nm and emission wavelength 460 nm using a Perkin–Elmer spectrofluorimeter (Wallac 1420 Explorer, Boston, MA, USA).

### 4.6. Measurement of Intracellular Calcium

A fluorometric assay was used to determine the intracellular calcium levels according to [27], with slight modification. RBL-2H3 cells were sensitized with 0.2 μg/mL of anti-DNP-IgE overnight. After washing with PBS three times, 4 μM Fluo 3-AM (containing 0.04% Pluronic F127) was loaded at 37 °C for 1 h. The cells were washed twice with PBS and harvested. RBL-2H3 cells (1 × 10^6^ /mL) were suspended and exposed to TIPP (50, 200, and 800 μg/mL) and ketotifen (20 μg/mL) in a PIPES buffer for 30 min. Then 1 μg/mL of DNP-HSA was added and the fluorescence intensity (FI) was determined every second at an excitation wavelength of 488 nm and emission wavelength of 525 nm with HITACHI F-7000 fluorescence spectrophotometer (Hitachi High-Tech, Tokyo, Japan).

### 4.7. Quantification PCR

RBL-2H3 cells were transferred into 12-well plates (1 × 10^6^ cells/well) and sensitized with 0.2 μg/mL of anti-DNP-IgE overnight. After triple washes and incubation with TIPP of different concentrations (0–800 μg/mL) for 30 min, the cells were challenged with 1 μg/mL of DNP-HSA for 1 h. Total RNAs was isolated using TRIzol reagent according to the manufacturer’s protocol. One microgram of total RNAs was used for the synthesis of cDNA using FastQuant RT Kit and target genes were assayed using SYBR^®^ Green Realtime PCR Master Mix (via Roche Light Cycler™) with their respective primers (Table 1). The PCR conditions were as follows: 95 °C for 30 s; 40 cycles of 95 °C for 5 s, 57 °C for 10 s, and 72 °C for 15 s. A transcription level of β-actin was used as an internal control to calculate fold induction and the fold changes in transcription levels were calculated using the 2^−ΔΔ*C*t^ method ΔΔ*C*t = (*C*t_target gene_ −* C*t_β-actin_)_treated groups_ − (*C*t_target gene_ − *C*t_β-actin_)_Normal_.

**Table 1:** Primers for quantification PCR of pro-inflammatory cytokine mRNAs in RBL-2H3 cells.

Genes	Forward Primer (5'→3')	Reverse Primer (5'→3')
*IL-3*	CCAGATTTCAGACAGGGGCTC	CAGGTTTACTCTCCGCAAGGT
*IL-4*	TCCACGGATGTAACGACAGC	TCATTCACGGTGCAGCTTCT
*IL-6*	CACTTCACAAGTCGGAGGCT	TCTGACAGTGCATCATCGCT
*IL-13*	ATGGTATGGAGCGTGGACCT	AGCGGAAAAGTTGCTTGGAG
*TNF-α*	ATGGGCTCCCTCTCATCAGT	GAAATGGCAAATCGGCTGAC
*MCP-1*	AGCCAACTCTCACTGAAGCC	AACTGTGAACAACAGGCCCA
*COX-2*	TGACTTTGGCAGGCTGGATT	ACTGCACTTCTGGTACCGTG
*β-actin*	GCATTGCTGACAGGATGCAG	GTAACAGTCCGCCTAGAAGCA

### 4.8. Confocal Fluorescence Microscope Observation

For membrane ruffling experiments, 1 × 10^5^ RBL-2H3 cells were loaded into a confocal dish and sensitized with 0.2 μg/mL of anti-DNP-IgE overnight [28]. After triple washes and incubation with TIPP of different concentrations (50, 200 and 800 μg/mL) for 30 min, the cells were challenged with 1 μg/mL of DNP-HSA for 0.5 h. Cells were fixed with 4% paraformaldehyde solution for 10 min at room temperature. After permeabilization in 0.1% Triton X-100 (in PBS), cells were incubated with rhodamin-phalloidin for 40 min at room temperature for F-actin staining. Then the cells were washed three times and stored in blocking solution (glycerol–PBS, 1:1, *v*/*v*) at 4 °C until observed with a confocal microscope (Carl Zeiss, Jena, German).

For membrane binding experiments, cells were incubated with 200 μg/mL of FITC-TIPP for 30 min at 37 °C, 5% CO_2_. After three washes with PBS, the cells were fixed and permeabilized as previously described. Then the cells were incubated with Hoechst 33,342 for 20 min at room temperature to stain nuclei, followed by membrane staining with 1,1'-dioctadecyl-3,3,3',3'-tetramethylindocarbocyanine perchlorate (DiI) for 10 min. RBL-2H3 cells were prepared for confocal microscope observation with treatment the same as before.

### 4.9. Flow Cytometry Analysis

Flow cytometry analysis was used to determine the effects of concentration, temperature, and incubation time on the interaction between TIPP and RBL-2H3 cells [29]. RBL-2H3 cells were incubated with different concentrations of FITC-TIPP for 30 min at 37 °C, 5% CO_2_ for dose-dependent detection. To determine the time and temperature dependence, cells were incubated with 200 μg/mL of FITC-TIPP for different times (2, 10, 30, and 60 min) at 37 °C or at different temperatures (4, 25, and 37 °C) for 30 min. Then cells were collected and analyzed by flow cytometry on a FACSCalibur using CellQuest software (BD Biosciences, Franklin Lakes, NJ, USA).

### 4.10. Western Blot Analysis

RBL-2H3 cells were loaded onto 6-well plates (2 × 10^6^ cells/well in 2 mL) and sensitized with 0.2 μg/mL of anti-DNP-IgE overnight. After triple washes with PBS, the cells were pre-treated with TIPP for 30 min, then stimulated with 1 μg/mL of DNP-HSA for a certain time. Nuclear proteins and cell total proteins were isolated with a cell nuclear protein extraction kit or cell total protein extraction kit. Equal amounts (20 μg) of proteins were separated by 10% SDS-PAGE gels and transferred to polyvinylidene difluoride membranes. After blocking with 5% skim milk in TBST containing 0.1% Tween 20, the membranes were incubated with primary antibodies at 4 °C overnight. Then the membranes were washed and incubated with horseradish peroxidase (HRP)-conjugated secondary antibodies for 1 h at room temperature. The target proteins were visualized with chemiluminescent HRP substrate.

### 4.11. Statistical Analysis

Data were analyzed by one-way analysis of variance (ANOVA) followed by Dunnett’s post-test using SPSS 18.0 software (IBM, Armonk, NY, USA). Results were presented as means ± SD or SEM and differences were considered significant when *p* < 0.05.

## 5. Conclusions

In conclusion, our study indicated that TIPP suppressed degranulation in IgE-activated RBL-2H3 cells. TIPP inhibited the increase in intracellular calcium and rearrangement of F-actin, and also significantly attenuated the transcription of pro-inflammatory cytokines. The inhibitory activity of TIPP in IgE-mediated activation of RBL-2H3 cells was regulated by MEK/ERK and NF-κB signaling pathways.
